# The Changing Pattern of Upper Gastrointestinal Disorders by Endoscopy: Data of the Last 40 Years 

**DOI:** 10.1155/2014/262638

**Published:** 2014-09-08

**Authors:** Erkan Caglar, Birol Baysal, Ahmet Dobrucalı

**Affiliations:** ^1^Department of Gastroenterology, Cerrahpasa Medical Faculty, Istanbul University, 34098 Istanbul, Turkey; ^2^Division of Gastroenterology, Department of Internal Medicine, Cerrahpasa Medical Faculty, Istanbul University, Cerrahpaşa Tıp Fakültesi, Fatih, 34098 Istanbul, Turkey

## Abstract

*Objectives*. We have investigated the changes in the incidence of various diagnoses that have been made in the endoscopy unit throughout the last 40 years.* Methods*. In this study, changes in the incidence of endoscopic diagnosis in upper gastrointestinal system between 1970 and 2010 were evaluated. Their diagnosis, age, and gender data were entered into the Excel software. *Results*. Of the 52816 cases who underwent esophagogastroduodenoscopy in the 40-year time period, the mean age was 48.17 ± 16.27 (mean ± SD). Although overall more than half of the patients were male (54.3%), in 1995 and after a marked increase was seen in the proportion of female gender (51–55%). The presence of hiatal hernia, reflux esophagitis, and the number of Barrett's esophaguses significantly increased. Erosive gastritis showed gradual increase, while the number of gastric ulcers decreased significantly. The presence of gastric and esophageal cancer significantly decreased. The number of duodenal ulcers significantly decreased. *Conclusion*. We detected that the incidences of esophagitis, Barrett's esophagus, and erosive gastritis significantly increased while the incidences of gastric/duodenal ulcer and gastric/esophageal cancer decreased throughout the last 40 years.

## 1. Introduction

During the last few decades, a change has been observed in the incidence of many gastrointestinal diseases, such as gastric cancer, acid-peptic disease including peptic ulcer, and gastroesophageal reflux disease [1]. Gastroesophageal reflux disease (GERD) was previously thought to be a rare disease in the East, but several recent reviews have also brought up the possibility of an increase in the prevalence of GERD. Esophagitis prevalence is reported to be 14.5% to 16.1% in patients for whom upper gastrointestinal endoscopy is performed due to dyspepsia and reflux [2–4]. Over the past three to four decades a decline in the prevalence of peptic ulcer disease in the West has been reported [5]. Similar observations have been made in the Asian-Pacific region as well [6]. The epidemiology of esophageal cancer has changed substantially over the last 50 years. It is a development that will certainly give rise to great concern. While the burden of gastric cancer remains high in the Asian Pacific region, age-standardized incidence rates have started to decline. This keeps up with observed trends in Western countries where gastric cancer has been observed to have declined since the 1940s [7].

Therefore, we retrospectively investigated the results of upper gastrointestinal system (GIS) endoscopy which was performed throughout the last 40 years.

## 2. Material and Methods

Istanbul is the most populated city in Turkey and its population has risen significantly during the last 40 years. Our faculty is a tertiary care institution. To determine the change of frequency of diagnosis in the upper gastrointestinal system, we retrospectively evaluated esophagogastroduodenoscopy (EGD) data recorded between the years 1970 and 2010 in the endoscopy laboratory of gastroenterology department. We reviewed 106 registries which were endoscopy reports performed between the 1970s and 2000s. We obtained the data from the year 2000 and thereafter from the computerized registries. From 2000 and on, a customized version of MedGate system from Aura was used. 56.652 data were reviewed and, of them, 52.856 were included in this study. Inadequate endoscopic reports were excluded from the study. The patients were grouped by 5-year periods. Their diagnosis, age, and gender information were recorded. After the registration of all diagnoses, it was simplified to general diagnoses and rarely seen endoscopic diagnoses. Many patients with a gastric ulcer underwent a follow-up gastroscopy a few months later. These follow-up endoscopies were excluded from the analysis. All included cases were newly diagnosed ulcers in a previously uninvestigated dyspeptic population.

For the endoscopies performed in the 1970s, the devices Olympus, JF-B2, GIF-D, K, and P2 were used. Between the years 1980 and 2000, the endoscopies were performed using the devices Olympus Fiberoptic GIF T10, Q10, and K10. In the 2000s, with the introduction of video camera systems, Pentax upper GIS endoscopy devices were used. Endoscopies were done on the request of a general practitioner or a specialist, mostly an internist or a gastroenterologist, sometimes a surgeon or a cardiologist. Biopsy samples were taken to confirm the macroscopic diagnosis if required.

Data is described as the mean ± standard deviation (SD). The frequency of upper gastrointestinal disorders was expressed in percentage. Statistical analysis was performed using Excel software.

## 3. Results

**Figure 1:**
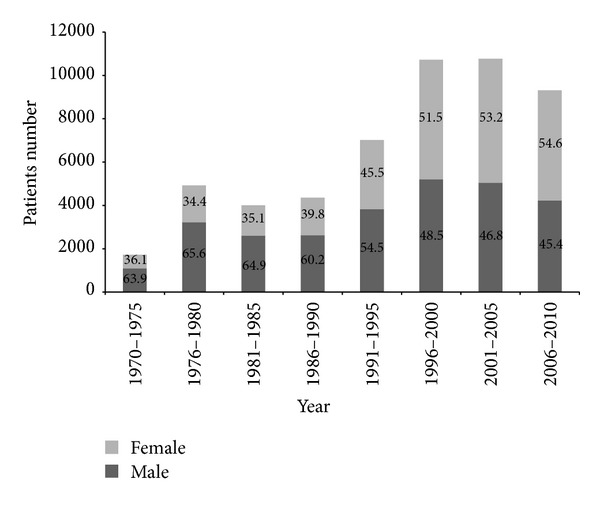
The distribution of the number of patients. Two colored bars show the percentage of males and females in the consecutive 5-year periods.

**Figure 2:**
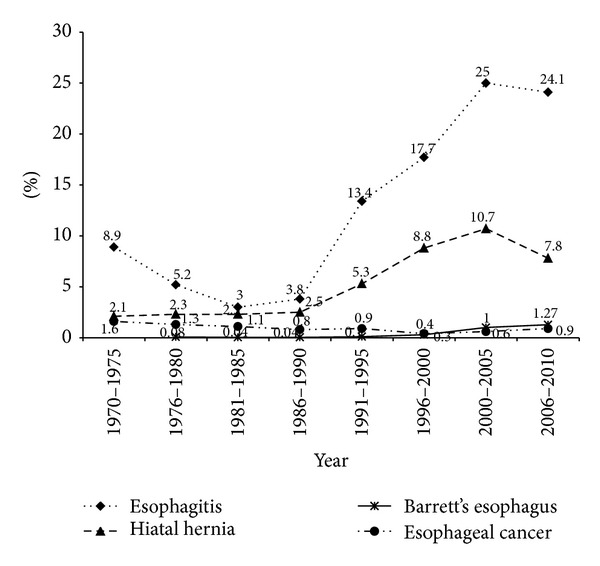
Changing of the prevalence of esophageal disease.

**Figure 3:**
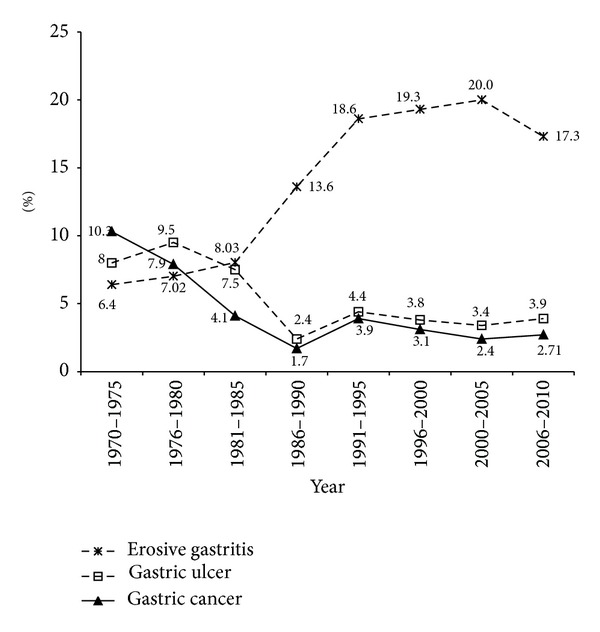
Relevant endoscopic diagnosis of gastric disease in the consecutive years' period.

**Figure 4:**
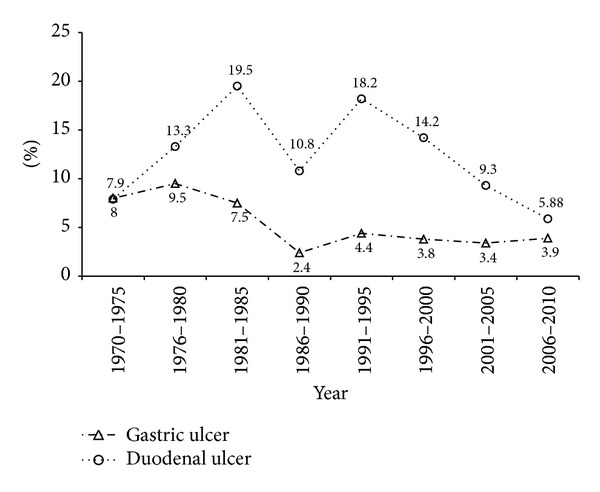
Changing of the prevalence of peptic ulcer disease.

Of the 52.856 cases who underwent EGD in the 40-year time period, the mean age was 48.17 ± 16.27 (mean ± SD). During the 5-year periods reviewed, a gradual increase of endoscopic examinations was observed (Figure 1). Figure 1 shows the percentage of numbers of men and women. Although overall more than half of the patients were male (54.3%), a marked increase was seen in the proportion of female gender (51–55%) in year 1995 and after. The total number of endoscopic diagnoses exceeds the total number of patients because in some patients multiple diagnoses were made.  Table 1 provides the numbers and the percentages of the principal diagnosed upper GIS pathologies by years. Figures 2, 3, and 4 show the change in frequency of different endoscopic diagnoses in time periods.

## 4. Discussion

Upper gastrointestinal endoscopy is an accurate and safe method to evaluate the mucosa of the esophagus, stomach, and duodenum. It is performed for a variety of indications, especially for diagnostic purposes. Among the gastrointestinal diseases, major changes have been observed in gastric and esophageal cancer, as well as with acid-peptic diseases including peptic ulcer and GERD. In our retrospective evaluation, we observed a marked increase in the incidence of esophagitis, Barrett's esophagus, gastritis, and bulbitis and a decrease in the incidence of duodenal ulcer, gastric ulcer, gastric cancer, and esophageal cancer.

In recent years the number of upper gastrointestinal endoscopies performed on the request of the general practitioner significantly increased in Turkey. The explanation is not only the presence of an open access facility, but also the more prominent place of gastroscopy in the work of dyspepsia and reflux disease [8]. Beside this, the number of women undergoing upper gastrointestinal endoscopy steadily increased in the consecutive years. Socioeconomic improvements have let women benefit from health services. Every Turkish citizen has a mandatory health insurance and hence an accessible health care.

Gastroesophageal reflux disease is a common problem in the West: among patients undergoing esophagoduodenoscopy for a variety of upper gastrointestinal symptoms, 9–23% had endoscopic esophagitis [9, 10]. Also recent studies from some parts of Asia have documented a prevalence of endoscopic esophagitis of up to 14.5% in patients evaluated for upper gastrointestinal tract symptoms [4]. Our study clearly shows that the incidence of endoscopic esophagitis increased over time. The increase may be due to altered nutritional habits, increased body mass index, and a declining rate of* Helicobacter pylori* (*H. pylori*) infection. Although a decreased incidence of* H. pylori* infection during the childhood period has been reported in Turkey [11], there is no data showing decreased incidence of infection in adult population. The increased attention paid to the lower esophagus during endoscopy may also contribute to the increase in the prevalence of reflux esophagitis. There is no doubt that the presence of hiatal hernia contributes to the occurrence of gastroesophageal reflux, which can lead to erosive esophagitis and Barrett's esophagus. We found that the presence of hiatal hernia was significantly increased over the periods in our series.

Barrett's esophagus (BE), a metaplastic condition caused by chronic gastroesophageal reflux, predisposes to adenocarcinoma of the esophagus. Data about the change in the incidence of Barrett's esophagus are conflicting. Todd et al. showed a decrease in reflux esophagitis and an increase in Barrett's esophagus in patients undergoing endoscopy in the period from 1980 to 1995 in Scotland [12]. On the contrary, Loffeld and Van Der Putten reported that esophagitis gradually increased but Barrett's esophagus remained stable during the last 10 years [13]. In our series, Barrett's esophagus significantly increased over the time, consistent with an increased background of reflux disease. Improvement in the endoscopic diagnosis of Barrett's esophagus may be due to the increased attention paid to the lower esophagus.

There is no doubt that the epidemiology of esophageal cancer has changed substantially over the last 50 years, especially in the Western world. In the United States and Europe, overall rates of esophageal cancer as well as squamous cell carcinoma have been decreasing, while rates of adenocarcinoma have been on the rise [14, 15]. In the East, esophageal cancer is predominantly squamous cell in type and there has not been a noticeable rise in the incidence of adenocarcinoma of the esophagus [16]. Fernandes et al. reported that the overall incidence of esophageal cancer has declined significantly in the multiethnic Singapore over the last 35 years [17]. The decrease is mainly a result of a steep decline in the incidence of squamous cell carcinoma (SCC), which is not offset by the marginal increase in the incidence of adenocarcinoma [12]. Similarly, Gholipour and colleagues reported that the incidence of overall esophageal cancer and squamous cell carcinoma has been declining during the years of their study [18]. Although the lack of knowledge about the histological subtypes limits our ability to make further comments, our results suggest that the frequency of overall esophageal cancer has been declining during the years of study. The decreased incidence of esophageal carcinoma may be attributed to the decreased consumption of traditional dried foods and improved sanitation. Although the decreased risk of squamous cell carcinoma is attributed to the decreased frequency of smoking in Western countries, this reason cannot explain the decrease seen in our study because the frequency of smoking has gradually increased during the last 50 years in Turkey. On the other hand, a conflict arises between decreased esophageal cancer and increased Barrett's esophagus in the last 15 years. The widespread using of proton-pump inhibitors in reflux disease may be a reason to some extent.

Peptic ulcer (PU) disease is believed to be less common and less severe as a result of modern medical treatment [19]. By El-Serag and Sonnenberg, a study that covers a 25-year period was performed in the United States. This study reported that the incidence of peptic ulcer had a marked decrease [5]. In the United Kingdom, Bardhan et al. reported that, during a 28-year period, the incidence of PU decreased, but a very slight decrease of the presentation to emergency services accompanied this [19]. Compared to the 1970s, we have identified a significant decrease of the incidence of peptic ulceration in the 2000s. In detailed retrospective evaluation, we found an increase in the incidence of PU during the period of 1970–1980. This increase may be due to the introduction and the common prescription of nonsteroidal anti-inflammatory drugs (NSAIDs) during these years. After the marketing of the first H2-receptor antagonists (H2RA) in the eighties a temporal consistency with the decrease was observed in PU incidence in Turkey. We observed a second decrease in the incidence of peptic ulcer disease during the period after 1995. The obvious explanation for this observation is the installment of the anti-*Helicobacter pylori* therapy, which is generally used in the cases of ulcer disease since 1993. Another explanation for the decreasing numbers of ulcers is the decreasing acquisition of* H. pylori*. In our study, the incidence of gastric ulcer showed an important decrease in the early 90s compared to the 70s. However, the decrease observed in the incidence of GU remained stable during the 1990s. This stability observed in the incidence of GU may be attributed to the increased population of elderly people and the increased usage of NSAIDs and aspirin. In their study performed in the 1990s in Australia, Xia et al. reported a decrease in the incidence of peptic ulcer, especially gastric ulcer (GU), but no marked decrease was reported for duodenal ulcer (DU) [20].

Gastritis is a heterogeneous pathological condition. According to published data the prevalence of gastritis among adults in the Western world changes between 37% and 62% [21, 22]. In the Zaanstreek (in The Netherlands) population, Loffeld et al. reported that erosive gastritis showed a gradual decrease in a period of 20 years after 1991. The reason for this was proposed to be due to the decrease of* H. pylori* incidence by some authors [23]. However, when we analyzed our series, we found that there was an increase of the diagnosis of erosive gastritis. The increase in NSAID use in recent years could be the reason for the increase in our series. In addition, this increase may be due to the introduction of new endoscopes with a higher resolution and the awareness of endoscopists for endoscopic gastritis increased after the introduction of Sydney classification [24].

Epidemiologically, the mortality rate for gastric cancer has decreased worldwide in the past several decades. It was reported that the incidence and the mortality of gastric cancer have gradually decreased in the Baltic Republic during the last four decades [25]. Miyahara et al. reported that the incidence of gastric cancer has gradually decreased in Japan during the last 30 years [26]. Consistent with other series, our series showed a gradual decrease in the incidence of gastric cancer. This decrease may be attributed to the alteration of dietary habits (the consumption of Western diets with low amount of nitrate), socioeconomic improvement, and the decrease of incidence of* H. pylori* infection. Along with the decreasing of the infection with* H. pylori*, changing incidence of chronic gastritis and changing of the diet may reflect the change observed in the incidence of gastric cancer [27, 28]. Although Turkey reported a decreased incidence of infection with* H. pylori* during the childhood period, high incidence of* H. pylori* and rate of failure in the eradication did not support the decrease of gastric cancer in our country [11, 29].

The limitations of this study are that the subjects were studied in a single hospital only. In addition, the fact that our hospital is a tertiary care institution may contribute to underestimation of real incidences of upper gastrointestinal pathologies in general population. The results may be influenced by the fact that, in the past, many different endoscopists, with different levels of experiences, worked at our center. The improvements in endoscopy technology and the changes of the definitions of endoscopic diagnosis (e.g., Barrett's esophagus) that occurred during that period naturally influenced the results.

## Figures and Tables

**Table 1:** Numbers and percentages of the principal endoscopic diagnoses made during the 40-year examination separated into 5-year periods (the disproportion of the numbers and of the percentages is due to more than one diagnosis made in one patient).

	1970–75	1976–80	1981–85	1986–90	1991–95	1996–00	2001–05	2006–10
Diagnosis *n* (%)	1727	4925	4007	4357	7018	10718	10772	9312

Esophagitis	154 (8.9%)	257 (5.2%)	119 (3.0%)	168 (3.8%)	943 (13.4%)	1897 (17.7%)	2964 (25.0%)	2247 (24.1%)
Hiatal hernia	36 (2.1%)	114 (2.3%)	94 (2.3%)	107 (2.5%)	397 (5.3%)	938 (8.8%)	1159 (10.7%)	733 (7.8%)
Barrett's esophagus	— (—)∗	4 (0.08%)	2 (0.04%)	2 (0.04%)	8 (0.1%)	33 (0.3%)	109 (1.0%)	119 (1.27%)
Esophagus cancer	27 (1.6%)	66 (1.3)	45 (1.1%)	37 (0.8%)	65 (0.9%)	44 (0.4%)	69 (0.6%)	89 (0.9%)
Erosive gastritis	112 (6.4%)	346 (7.02%)	322 (8.03%)	595 (13.6%)	1307 (18.6%)	2072 (19.3%)	2160 (20.0%)	1617 (17.3%)
Gastric ulcer	138 (8.0%)	470 (9.5%)	300 (7.5%)	105 (2.4%)	310 (4.4%)	409 (3.8%)	366 (3.4%)	366 (3.9%)
Gastric cancer	178 (10.3%)	391 (7.9%)	166 (4.1%)	74 (1.7%)	277 (3.9%)	330 (3.1%)	255 (2.4%)	253 (2.71%)
Duodenal ulcer	136 (7.9%)	657 (13.3%)	782 (19.5%)	474 (10.8%)	1278 (18.2%)	1522 (14.2%)	1002 (9.3%)	548 (5.88%)
Normal endoscopic findings	290 (16.7%)	552 (11.2%)	325 (8.1%)	530 (12.1%)	2846 (40.5%)	5524 (51.5%)	4927 (45.7%)	5296 (56.8%)

*In this period of examination, no diagnosis of Barrett's esophagus was made.

## References

[1] Goh K-L, Wong H-T, Lim C-H, Rosaida MS (2009). Time trends in peptic ulcer, erosive reflux oesophagitis, gastric and oesophageal cancers in a multiracial Asian population. *Alimentary Pharmacology and Therapeutics*.

[2] Shaheen N, Provenzale D (2003). The epidemiology of gastroesophageal reflux disease. *The American Journal of the Medical Sciences*.

[3] Sarıoğlu M, Kabaçam G, Bektas M, et al (2009). The change in esophagitis detection rates during the last two decades at an endoscopy cented. *Endoskopi*.

[4] Yeh C, Hsu C-T, Ho A-S, Sampliner RE, Fass R (1997). Erosive esophagitis and Barrett's esophagus in Taiwan: a higher frequency than expected. *Digestive Diseases and Sciences*.

[5] El-Serag HB, Sonnenberg A (1998). Opposing time trends of peptic ulcer and reflux disease. *Gut*.

[6] Wong SN, Sollano JD, Chan MM (2005). Changing trends in peptic ulcer prevalence in a tertiary care setting in the Philippines: a seven-year study. *Journal of Gastroenterology and Hepatology*.

[7] Borch K, Jönsson B, Tarpila E (2000). Changing pattern of histological type, location, stage and outcome of surgical treatment of gastric carcinoma. *The British Journal of Surgery*.

[8] Loffeld RJ, van der Putten AB (2003). The yield of UGIE: a study of a ten-year period in the “Zaanstreek”. *The Netherlands Journal of Medicine*.

[9] Fjøsne U, Kleveland PM, Waldum H, Halvorsen T, Petersen H (1986). The clinical benefit of routine upper gastrointestinal endoscopy. *Scandinavian Journal of Gastroenterology*.

[10] Stoker DL, Williams JG, Leicester RG, Colin-Jones DG (1988). Oesophagitis: a five year review. *Gut*.

[11] Özden A, Bozdayi G, Özkan M, Köse KS (2004). Changes in the seroepidemiological pattern of Helicobacter pylori infection over the last 10 years in Turkey. *Turkish Journal of Gastroenterology*.

[12] Todd JA, Johnston DA, Dillon JF (2002). The changing spectrum of gastroesophageal reflux disease. *European Journal of Cancer Prevention*.

[13] Loffeld RJLF, Van Der Putten ABMM (2003). Rising incidence of reflux oesophagitis in patients undergoing upper gastrointestinal endoscopy. *Digestion*.

[14] Corley DA, Buffler PA (2001). Oesophageal and gastric cardia adenocarcinomas: analysis of regional variation using the cancer incidence in five continents database. *International Journal of Epidemiology*.

[15] Pera M, Manterola C, Vidal O, Grande L (2005). Epidemiology of esophageal adenocarcinoma. *Journal of Surgical Oncology*.

[16] Law S, Wong J (2002). Changing disease burden and management issues for esophageal cancer in the Asia-Pacific region. *Journal of Gastroenterology and Hepatology*.

[17] Fernandes ML, Seow A, Chan Y-H, Ho K-Y (2006). Opposing trends in incidence of esophageal squamous cell carcinoma and adenocarcinoma in a multi-ethnic Asian country. *The American Journal of Gastroenterology*.

[18] Gholipour C, Shalchi RA, Abbasi M (2008). A histopathological study of esophageal cancer on the western side of the Caspian littoral from 1994 to 2003. *Diseases of the Esophagus*.

[19] Bardhan KD, Williamson M, Royston C, Lyon C (2004). Admission rates for peptic ulcer in the Trent Region, UK, 1972–2000. Changing pattern, a changing disease?. *Digestive and Liver Disease*.

[20] Xia HH-X, Phung N, Altiparmak E, Berry A, Matheson M, Talley NJ (2001). Reduction of peptic ulcer disease and Helicobacter pylori infection but increase of reflux esophagitis in Western Sydney between 1990 and 1998. *Digestive Diseases and Sciences*.

[21] Dooley CP, Cohen H, Fitzgibbons PL (1989). Prevalence of helicobacter pylori infection and histologic gastritis in asymptomatic persons. *The New England Journal of Medicine*.

[22] Johnsen R, Bernersen B, Straume B, Førde OH, Bostad L, Burhol PG (1991). Prevalences of endoscopic and histological findings in subjects with and without dyspepsia. *British Medical Journal*.

[23] Loffeld RJLF, Liberov B, Dekkers PEP (2012). The changing prevalence of upper gastrointestinal endoscopic diagnoses: a single-centre study. *The Netherlands Journal of Medicine*.

[24] Misiewicz JJ, Tytgat GNJ, Goodwin CS The Sydney system: a new classification of gastritis. Working Party Reports.

[25] Klint Å, Engholm G, Storm HH (2010). Trends in survival of patients diagnosed with cancer of the digestive organs in the Nordic countries 1964–2003 followed up to the end of 2006. *Acta Oncologica*.

[26] Miyahara R, Niwa Y, Matsuura T (2007). Prevalence and prognosis of gastric cancer detected by screening in a large Japanese population: data from a single institute over 30 years. *Journal of Gastroenterology and Hepatology*.

[27] Kim MK, Sasaki S, Sasazuki S, Tsugane S (2004). Prospective study of three major dietary patterns and risk of gastric cancer in Japan. *International Journal of Cancer*.

[28] Tokui N, Yoshimura T, Fujino Y (2005). Dietary habits and stomach cancer risk in the JACC study. *Journal of Epidemiology*.

[29] Kadayifci A, Buyukhatipoglu H, Savas MC, Simsek I (2006). Eradication of *Helicobacter pylori* with triple therapy: an epidemiologic analysis of trends in Turkey over 10 years. *Clinical Therapeutics*.

